# Real-World Data in Pharmacovigilance Database Provides a New Perspective for Understanding the Risk of *Clostridium difficile* Infection Associated with Antibacterial Drug Exposure

**DOI:** 10.3390/antibiotics12071109

**Published:** 2023-06-27

**Authors:** Dongxuan Li, Yi Song, Zhanfeng Bai, Xin Xi, Feng Liu, Yang Zhang, Chunmeng Qin, Dan Du, Qian Du, Songqing Liu

**Affiliations:** 1 Department of Pharmacy, The Third Affiliated Hospital of Chongqing Medical University, Chongqing 401120, China; 2 College of Pharmacy, Chongqing Medical University, Chongqing 400016, China; 3 Center for Medical Information and Statistics, The Third Affiliated Hospital of Chongqing Medical University, Chongqing 401120, China; 4 Medical Data Science Academy, Chongqing Medical University, Chongqing 400016, China

**Keywords:** *Clostridium difficile* infection, antibacterial drug, FDA Adverse Event Reporting System, pharmacovigilance, disproportionality analysis, adverse reaction

## Abstract

Antibacterial drug exposure (ADE) is a well-known potential risk factor for *Clostridium difficile* infection (CDI), but it remains controversial which certain antibacterial drugs are associated with the highest risk of CDI occurrence. To summarize CDI risk associated with ADE, we reviewed the CDI reports related to ADE in the FDA Adverse Event Reporting System database and conducted disproportionality analysis to detect adverse reaction (ADR) signals of CDI for antibacterial drugs. A total of 8063 CDI reports associated with ADE were identified, which involved 73 antibacterial drugs. Metronidazole was the drug with the greatest number of reports, followed by vancomycin, ciprofloxacin, clindamycin and amoxicillin. In disproportionality analysis, metronidazole had the highest positive ADR signal strength, followed by vancomycin, cefpodoxime, ertapenem and clindamycin. Among the 73 antibacterial drugs, 58 showed at least one positive ADR signal, and ceftriaxone was the drug with the highest total number of positive signals. Our study provided a real-world overview of CDI risk for AED from a pharmacovigilance perspective and showed risk characteristics for different antibacterial drugs by integrating its positive–negative signal distribution. Meanwhile, our study showed that the CDI risk of metronidazole and vancomycin may be underestimated, and it deserves further attention and investigation.

## 1. Introduction

*Clostridium difficile* is an anaerobic, spore-forming Gram-positive bacillus that usually colonizes in the human gut [1]. It is an opportunistic pathogen that is able to abnormally proliferate, produce toxins and result in diarrhea, especially in patients with changes in the indigenous colonic microbiota following antibiotic use [2], and it is reported that the attributable mortality of *C. difficile* infection (CDI) should be at least 5.99% [3]. In recent decades, the increasing incidence, severity and mortality of CDI have made it a challenging clinical problem for medical personnel [4]. In response to this challenge, diagnosis and treatment guidelines have been developed in recent years to optimize the management of CDI [5,6,7,8,9]. In primary prevention for CDI, the careful selection of antibacterial drugs and, whenever possible, the avoidance of high-risk antibacterial drug exposure (ADE) is the mainstay because most cases of CDI are both iatrogenic and nosocomial [4]. Meanwhile, some studies have shown that strict antimicrobial stewardship is beneficial in reducing CDI rates [10,11,12], which also demonstrated the need to understand the CDI risk of different antibacterial agents to formulate management strategies. However, although it is well known that antibacterial therapy plays a central role in the pathogenesis of CDI [2,13], it remains controversial whether certain antibacterial drugs or classes of antibacterial drugs are potentially associated with an increased risk of CDI [14,15]. Therefore, there is a need to assess the potential risk of CDI caused by different antibacterial drugs with a uniform metric.

Currently, pharmacovigilance databases are widely used for real-world post-marketing studies and as a tool to summarize the real-time safety profile of medical products to provide information for clinical practice [16]. In pharmacovigilance practice, according to finding disproportionality between drug usage and adverse events (AEs) occurrence in the pharmacovigilance databases, these real-world AEs data can provide a reference for identifying the potential culprit drugs of specific AE, optimizing the drug selection for individual patients and exploring the interaction between drugs [17]. In terms of exploring the safety profile of antibiotics by using the pharmacovigilance database, Seo, H. and Kim, E. elaborated on the risk characteristics of electrolyte disorders associated with piperacillin/tazobactam and detected the significant signal of hypokalemia for piperacillin/tazobactam compared with other penicillins [18]; Patek, T.M. et al. investigated acute kidney injury reports related to antibiotics in the FDA Adverse Event Reporting System (FAERS) database and found 14 classes of antibiotics that were significantly associated with acute kidney injury [19]. CDI is a representative AE associated with ADE, so real-world AE information in pharmacovigilance databases can provide an unprecedented opportunity to understand the potential risk of CDI caused by different antibacterial drugs.

In this study, we summarized the report characteristics of antibacterial drug-associated CDI cases in the FAERS database and evaluated the statistical connection between ADE and CDI occurrence by using a well-established adverse reaction (ADR) signal detecting method, trying to distinguish the risk of CDI induced by different antibacterial drugs from the pharmacovigilance perspective, so as to provide a reference for better primary prevention for CDI and antimicrobial stewardship.

## 2. Results

### 2.1. Report Basic Information and Patient Characteristics

**Figure 1:**
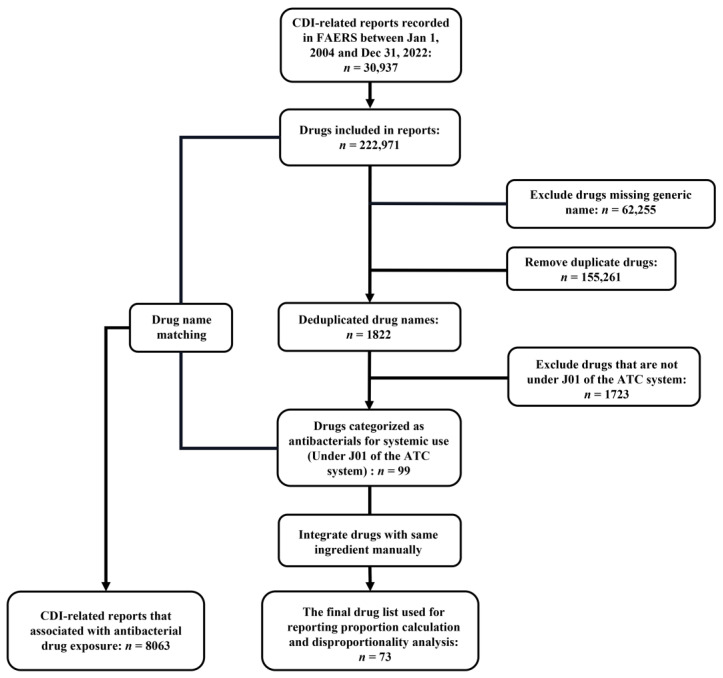
Flowchart of target reports identification. Abbreviations: ATC, Anatomical Therapeutic Chemical classification.

A total of 16,010,899 reports were recorded in the FAERS database from 1 January 2004 to 31 December 2022. Using the Preferred Terms (PTs) in Table 1 to retrieve target reports, a total of 30,937 reports considered CDI-related were returned and downloaded. As the culprit drug of CDI may be indecisive and can be attributed to multiple drugs, there were a total of 222,971 drugs contained in those CDI-related reports. After excluding drugs missing generic names, duplicated drugs and drugs that were not under J01 of the Anatomical Therapeutic Chemical (ATC) classification system, a total of 99 drug names were classified into “antibacterials for systemic use (J01)”. The 99 drug names were used to match reports that CDI occurrence was related to antibacterial drug use, and finally, a total of 8063 (26.1%) reports were identified for further analysis. As some of the 99 drug names were synonymous (e.g., ampicillin and ampicillin sodium), we integrated drugs with the same ingredient manually, and finally, there were 73 drugs included in the final antibacterial drug list to detect ADR signals. The detailed processing flow is shown in Figure 1.

**Figure 2:**
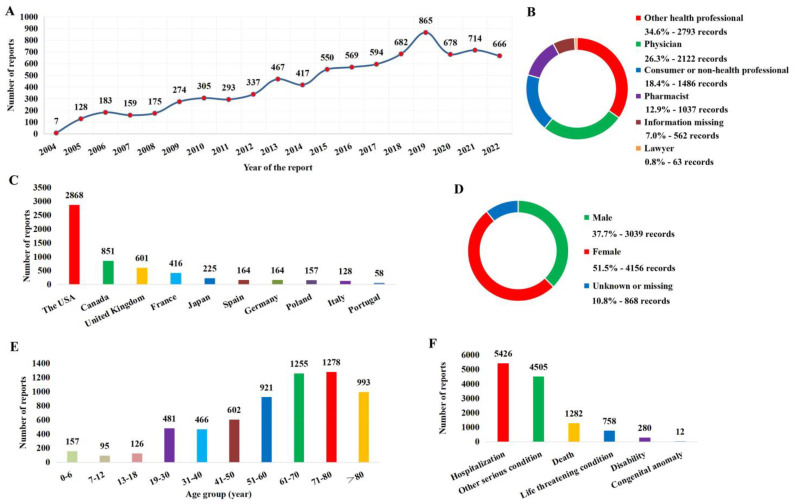
Report basic information and patient characteristics. (**A**) Distribution of the reporting year. (**B**) Distribution of reporter. (**C**) The top 10 countries with the most sources of reports. (**D**) Distribution of patient gender. (**E**) Distribution of patient age. (**F**) Distribution of patient outcome.

Information in the 8063 antibacterial drug use-related CDI reports was extracted and collected. The annual number of reports from 2004 to 2022 was presented in Figure 2A, among which 2019 was the year that FAERS received the greatest number of CDI reports associated with ADE. With regard to report sources, health professionals (73.8%) were the main submitters (Figure 2B), and the USA was the leading reporting country (Figure 2C). The demographic characteristics of patients were summarized, and the result showed that there were fewer male patients than female patients (Figure 2D) and the age of those patients was mainly located in the 71–80 age group (Figure 2E). In terms of patient outcome, CDI usually resulted in hospitalization (67.3%), and even the death of 1282 (15.9%) patients were associated with CDI (Figure 2F).

### 2.2. ADR Signal Detection Results

After integrating synonymous drugs, 73 antibacterial drugs were used to detect ADR signals at Standardized MedDRA Queries (SMQ) level and PT level. The signal detection results at the SMQ level are shown in Table 2, and it showed that metronidazole (*a* = 2004) was the most reported antibacterial drug followed by vancomycin (*a* = 1793), ciprofloxacin (*a* = 1176), clindamycin (*a* = 823) and amoxicillin (*a* = 566), while metronidazole (*ROR* = 22.10, 95% *CI* 21.10–23.14) had the highest positive signal strength followed by vancomycin (*ROR* = 21.30, 95% *CI* 20.29–22.36), cefpodoxime (*ROR* = 19.26, 95% *CI* 13.02–28.49), ertapenem (*ROR* = 16.69, 95% *CI* 14.30–19.49) and clindamycin (*ROR* = 16.29, 95% *CI* 15.18–17.47). In addition, the signal detection results for 10 different PT levels are shown in Tables S1–S10.

As metronidazole and vancomycin were usually used as therapeutic agents for CDI, we further reviewed the indications for metronidazole and vancomycin recorded in the “patient.drug.drugindication” field. In order to eliminate the influence of this factor on ADR signal detection results as much as possible, if the indication of metronidazole and vancomycin was related to the treatment of CDI, the report was excluded. The adjusted signal detection results for metronidazole and vancomycin at the SMQ level and PT level are shown in Table 3 and Table 4, respectively.

### 2.3. Distribution of ADR Signals

**Figure 3:**
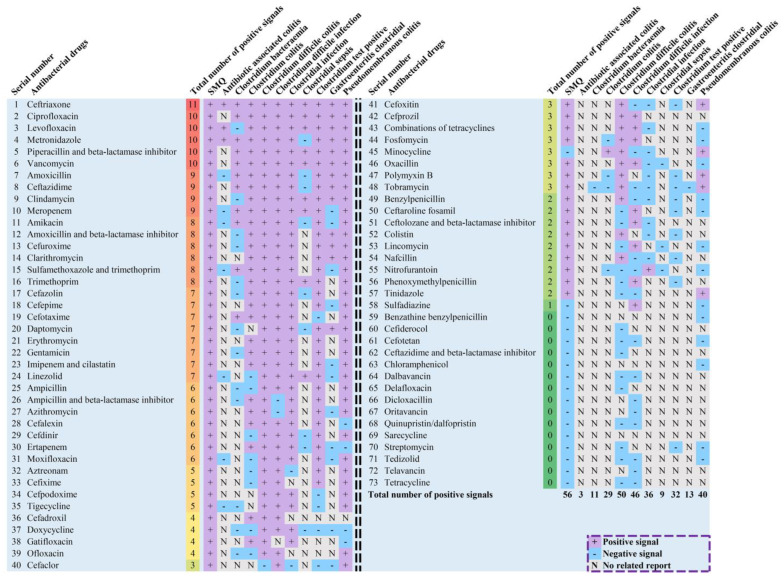
Pharmacovigilance signal distribution at the SMQ level and Preferred Term level. Note: the adjusted signal detection results for metronidazole and vancomycin at the SMQ level and PT level were used to show signal distribution. Abbreviations: SMQ, Standardized MedDRA Queries.

There were 11 ADR signal detection results for each of the 73 antibacterial drugs, including one for the SMQ level and 10 for the PT level. In addition, signal detection results can be divided into three states, namely positive signals, negative signals and not reported for target drug-AE combinations. The distribution of signal detection results for 73 antibacterial drugs is presented in Figure 3. It showed that 58 antibacterial drugs had at least one positive ADR signal detection, while another 15 antibacterial drugs did not show any positive signals at the SMQ level or PT level, although there were CDI cases reported. Of these antibacterial drugs with positive signals, only ceftriaxone had 11 positive signals.

## 3. Discussion

Antibacterial drugs, one of the greatest achievements of human beings in the field of medicine, have played an extremely important role in improving human health level and ensuring life safety. However, with the extensive use of antibacterial drugs in clinical practices, various ADRs associated with antibacterial drugs have emerged, among which CDI is one of the most noteworthy potentially life-threatening ADRs [20]. Therefore, it is necessary to determine the risk of CDI induced by different antibacterial drugs. In this study, we reviewed CDI reports associated with ADE in the FAERS database between 2004 to 2022 and found that 73 antibacterial drugs were recorded as potential culprit drugs. At the same time, based on the aforementioned antibacterial drug list, we conducted a disproportionality analysis to evaluate the risk correlation between the occurrence of CDI and ADE. As far as we know, this is the first study using a pharmacovigilance database to evaluate the risk of CDI occurrence for ADE.

Although it is widely recognized that any antimicrobial therapy increases the risk of CDI and there is a difference among different antibiotics, it remains controversial which certain antibiotics or classes of antibiotics are related to the highest risk of CDI. A previous study showed that fluoroquinolones were the antibacterial agent most strongly associated with CDI, while all the third-generation of cephalosporins, macrolides, clindamycin and intravenous beta-lactam/beta-lactamase inhibitors were intermediate-risk antibacterial agent [13]. Another study showed that the risk of hospital-acquired CDI was greatest for cephalosporins and clindamycin, while the importance of fluoroquinolones should not be overemphasized [21]. A recent study suggested that the highest-risk antibacterial agents related to CDI occurrence included second-generation and later cephalosporins, carbapenems, fluoroquinolones and clindamycin, while doxycycline and daptomycin were related to a lower CDI risk [22]. However, due to the difference in the region, patient inclusion and exclusion criteria, study design, drugs involved in the evaluation and the definition of risk classification, it is difficult to unify the CDI risk of antibacterial agents. In this regard, by using a unified standard to detect the ADR signals for each antibacterial agent at the PT and SMQ level, our study added new evidence for understanding the risk of CDI induced by ADE from a pharmacovigilance perspective. In comparison to the studies mentioned above, the advantage of this study is that it makes full use of real-world data to get a complete antibiotics list leading to CDI occurrence, involving 73 antibiotics commonly used in clinical settings. Therefore, our study can provide a more comprehensive overview of the risk of antibacterial drugs, facilitating a comparison of risks between them and providing a reference for antimicrobial stewardship.

Consistent with previous studies [13,21,22], our ADR signal detection results showed a high risk of CDI in most fluoroquinolones, cephalosporins, carbapenems, macrolides, clindamycin and beta-lactam/beta-lactamase inhibitors, which proved the credibility of our results to some extent. However, it is noteworthy that, in ADR signal detection, metronidazole and vancomycin have a surprising number of reports and high signal strengths at the SMQ level and PT level, although they have been reported as possible causes of CDI in previous studies [23,24]. There are several possible explanations for this noteworthy result. First, metronidazole and vancomycin were used as therapeutic drugs for CDI [5,6,7,8,9], which may lead us to ignore their risk of inducing CDI, while our research detected this neglected risk relationship. Second, 26.2% of reporters in this study were non-health professionals, so they may confuse therapeutic and etiological drugs and misjudge culprit drugs, which may result in biased results. Third, due to FAERS being a database with a voluntary reporting nature, underreporting of other antibacterial agents may exist [25], which may highlight the CDI risks of metronidazole and vancomycin. In order to reduce the influence of misreporting due to the overlap of indications and AEs for metronidazole and vancomycin, we excluded the reports that the indication of metronidazole and vancomycin was related to the treatment of CDI, but the adjusted signal detection results for metronidazole and vancomycin still showed conspicuous high potential risk (Table 3 and Table 4). In this regard, our results showed a warning that we should pay more attention to the CDI risk of metronidazole and vancomycin, which may have been previously neglected. Although the true relationship between CDI occurrence and vancomycin and metronidazole still needs a well-designed study to verify, we think there are two main potential values for these data. First, it provides evidence of the potential high risk of CDI induced by vancomycin and metronidazole, so it may help us identify previously neglected CDI cases induced by vancomycin and metronidazole. In this way, we can timely take measures, such as stopping taking medicine, changing medicine and etiological treatment, to protect patients from unnecessary sustained injury. Second, due to the potential high CDI risk signals of vancomycin and metronidazole, our results provided an opportunity to investigate further the CDI risks of vancomycin and metronidazole, which may affect future clinical practice in primary prevention of CDI and antimicrobial stewardship.

In addition to detecting ADR signals of 73 antibacterial drugs at the SMQ level and PT level and adjusting signal detection results for metronidazole and vancomycin, we also integrated the positive-negative distribution of their ADR signals, and the total number of positive signals was between 0 to 11 for each antimicrobial drug. If an antimicrobial drug has a relatively large total number of positive signals, it may mean that its risk of CDI is relatively high [26]. For example, in this study, ceftriaxone, one of the antimicrobial drugs belonging to the third generation of cephalosporins and one of the well-known high-CDI-risk antimicrobial agents, was the only drug showing 11 positive signals. In this regard, this indicator concisely summarized the CDI risk characteristics of antimicrobial drugs, facilitating to get a quick understanding of the risk of different antibacterial agents.

Although this study comprehensively summarized the CDI risk of antibacterial drugs by using a pharmacovigilance database, there were also some inevitable limitations in this study. First, due to the intrinsic limitations of the pharmacovigilance database, the fact that un-peer-reviewed data, underreporting, Weber effect and notoriety bias may lead to biased results [25,27,28]. Second, due to the total number of patients exposed to each antibacterial drug is unclear, the incidence of CDI for an antibacterial drug cannot be determined. Third, patient gender, age, concomitant therapeutic drugs, dose and duration of antibiotic use, and comorbidities may influence the occurrence of CDI, but it is almost impossible to shield the potential interference of those factors to our results due to the intrinsic limitations of the pharmacovigilance database. Fourth, the ADR signal result only represents the strength of the statistical association between the drug of interest and AE of interest, so a well-designed study is still needed to verify whether there is a true causality.

## 4. Materials and Methods

### 4.1. Data Source

The data in this study were obtained from the FAERS database, a large international pharmacovigilance database with voluntary reporting nature, which recorded ADRs information related to post-market, FDA-approved medications as well as natural substances, vaccines and medical devices [29]. It currently publicly opens more than 16 million drug post-marketing AEs records reported by manufacturers, consumers and healthcare professionals and updates quarterly. The recorded information in the database includes but is not limited to patient demographic information, report sources, medication information, AEs involved and patient outcomes [30]. Those data are highly structured and can be retrieved, collected and downloaded from the openFDA platform by constructing an appropriate retrieval statement through an application programming interface (API) [31]. In this study, we summarized and analyzed CDI reports related to ADE between 1 January 2004 and 31 December 2022 in FAERS.

### 4.2. Identification of CDI Reports Associated with Antibacterial Drug Use in FAERS

The FAERS reporting system uses the PTs in Medical Dictionary for Regulatory Activities (MedDRA) to standardize AEs occurring in patients [30]. SMQs are a series of PT sets that potentially indicate the same medical condition, which was developed to optimize data retrieval and signal detection in pharmacovigilance activity [32]. Within an SMQ, PTs can be further divided into narrow-scope PTs and broad-scope PTs according to the degree of association with the condition or area of interest [33]. Among them, the PTs with a narrow scope are closely related to the condition or area of interest, while such association is relatively weak for PTs with a broad scope.

Pseudomembranous colitis is an inflammatory condition of the colon characterized by the presence of yellow-white exudative plaques that coalesce to form pseudomembranes on the mucosa, and it is usually a marker of severe CDI [34]. Meanwhile, it is also one of the SMQs in MedDRA that includes many PTs potentially indicating CDI, so it can be used to identify CDI-related reports in FAERS. In order to improve the accuracy of case identification and signal detection, in this study, only narrow-scope PTs of pseudomembranous colitis (SMQ) in MedDRA 23.0 were selected to retrieve CDI-related reports in FAERS (Table 1). According to the ATC classification system, if one of the generic drug names recorded in the “patient.drug.openfda.generic_name” field can be classified into “antibacterials for systemic use (J01)” in a report, this report is considered CDI reports associated with ADE and included in the final analysis.

### 4.3. ADR Signal Detection Method

Disproportionality analysis is a kind of technology used to detect ADR signals at present. Based on the classical two-by-two contingency table (Table 5), researchers can compare the differences between the occurrence frequency and background frequency for target drugs and target AEs. The reporting odd ratio (*ROR*) is one of the well-established disproportionality analysis methods, which calculates the ratio of the odds of a selected drug versus all other drugs for a certain AEs compared to the odds of the same drugs for all other AEs recorded in FAERS to detect potential ADR signals [35]. In this study, we used the *ROR* and its corresponding 95% confidence intervals (CIs) to identify ADR signals, and the *ROR* and its 95% *CI* can be calculated by the following formula:

(1) $$ROR=\frac{a/c}{b/d}=\frac{ad}{bc},$$

(2) $$95\%\;CI=e^{\ln\left(ROR\right)\pm 1.96\sqrt{\left(\frac{1}{a}+\frac{1}{b}+\frac{1}{c}+\frac{1}{d}\right)}}.\;$$

When the lower-bound 95% *CI* of *ROR* was above 1.0 with at least three cases (*a* ≥ 3 in Table 5), it was considered a positive signal, suggesting a potential risk of the target AE caused by the target drug; instead, if the lower-bound 95% *CI* of *ROR* and the number of cases cannot meet the above-mentioned threshold, it was regarded as a negative signal, suggesting the statistical connection between target AE occurrence and target drug use is weak [26,36]. To some extent, the *ROR* value represents a statistical correlation between the drug of interest and AE of interest, and the *ROR* value is larger, the stronger the statistical correlation. Using this indicator, we can highlight the AE that may be induced by a certain drug and conduct a further investigation so as to inform the possible risk; on the other hand, we can also use it to compare the risk of different drugs causing the same AE, so as to guide the selection of therapeutic drugs or discontinuation of a culprit drug [17].

### 4.4. Data Collection and Analysis

With reference to the API build guideline issued by the openFDA (https://open.fda.gov/apis/drug/event/how-to-use-the-endpoint/, accessed on 1 January 2023), we can retrieve and download the target reports for further analysis. The specific data collection and analysis steps of this study are as follows.

Firstly, by using the R package “httr” to call API, PTs in Table 1 were used to retrieve target reports in FAERS, and the returned dataset was downloaded in “json” format. Secondly, the R package “jsonlite” was used to read the downloaded dataset and extract the reports information, including Safety Report ID number, patient demographics, report time, report sources, medication use and outcomes. Thirdly, generic drug names recorded in the “patient.drug.openfda.generic_name” field were used to further identify reports associated with antibacterial drug use, and report characteristics were summarized. Fourthly, the ADR signals at the SMQ level and PT level were detected by calculating the *ROR* value and its 95% *CI* by using disproportionality analysis, and 11 signals were generated for each antibacterial drug. Finally, the positive-negative distribution of signals was summarized.

In this study, R version 4.1.0 (R Foundation for Statistical Computing, Vienna, Austria) was used for data processing and analysis.

## 5. Conclusions

The vastness, authenticity and accessibility of FAERS data have made it an important resource for evaluating drug safety cost-effectively. In this study, CDI reports associated with ADE in FAERS were summarized, and the CDI risk of different antibacterial agents was explored. As the first study to evaluate CDI risk related to antibacterial drug exposure using a pharmacovigilance database, our study provided a preliminary picture of CDI induced by antibacterial drugs in the real world that can help to better primary prevention for CDI and antimicrobial stewardship. Meanwhile, the potentially high CDI risk of metronidazole and vancomycin that may have been previously overlooked was detected, and it deserved further attention from regulators, health professionals and others involved in antimicrobial stewardship. Of particular note, however, our study as a pharmacovigilance study using the FAERS database only provided a statistical association between CDI occurrence and antibacterial drugs, so further well-designed study is still necessary to validate the causality.

## Figures and Tables

**Table 1:** The narrow PT included in Standardized MedDRA Queries of pseudomembranous colitis.

PT	MedDRA Code
Antibiotic associated colitis	10052815
Clostridium bacteraemia	10058852
Clostridium colitis	10058305
Clostridium difficile colitis	10009657
Clostridium difficile infection	10054236
Clostridial infection	10061043
Clostridial sepsis	10078496
Clostridium test positive	10070027
Gastroenteritis clostridial	10017898
Pseudomembranous colitis	10037128

Abbreviations: PT, Preferred Term; MedDRA, Medical Dictionary for Drug Regulatory Activities.

**Table 2:** Pharmacovigilance signal detection results at the SMQ level.

Medication	Drug of Interest with AE of Interest (*a*)	Other Drugs with AE of Interest (*b*)	Drug of Interest with Other AEs (*c*)	Other Drugs with Other AEs (*d*)	*ROR* (95% *CI*)
**Tetracyclines (J01AA)**					
Doxycycline	227	30,710	46,700	15,933,262	2.52 (2.21–2.87)
Tigecycline	78	30,859	4193	15,975,769	9.63 (7.70–12.05)
Minocycline	32	30,905	12,056	15,967,906	1.37 (0.97–1.94)
Combinations of tetracyclines	17	30,920	816	15,979,146	10.77 (6.66–17.41)
Tetracycline	2	30,935	371	15,979,591	2.78 (0.69–11.18)
Sarecycline	1	30,936	105	15,979,857	4.92 (0.69–35.25)
**Amphenicols (J01BA)**					
Chloramphenicol	1	30,936	26	15,979,936	19.87 (2.70–146.41)
**Penicillins with extended spectrum (J01CA)**					
Amoxicillin	566	30,371	57,359	15,922,603	5.17 (4.76–5.62)
Ampicillin	88	30,849	6189	15,973,773	7.36 (5.96–9.09)
**Beta-lactamase sensitive penicillins (J01CE)**					
Phenoxymethylpenicillin	10	30,927	1592	15,978,370	3.25 (1.74–6.04)
Benzylpenicillin	8	30,929	1613	15,978,349	2.56 (1.28–5.13)
Benzathine benzylpenicillin	1	30,936	559	15,979,403	0.92 (0.13–6.57)
**Beta-lactamase resistant penicillins (J01CF)**					
Oxacillin	10	30,927	893	15,979,069	5.79 (3.10–10.79)
Nafcillin	10	30,927	802	15,979,160	6.44 (3.45–12.02)
Dicloxacillin	1	30,936	116	15,979,846	4.45 (0.62–31.88)
**Combinations of penicillins, incl. beta-lactamase inhibitors (J01CR)**					
Piperacillin and beta-lactamase inhibitor	483	30,454	19,305	15,960,657	13.11 (11.97–14.36)
Amoxicillin and beta-lactamase inhibitor	326	30,611	21,834	15,958,128	7.78 (6.97–8.69)
Ampicillin and beta-lactamase inhibitor	51	30,886	2129	15,977,833	12.39 (9.39–16.36)
**First-generation cephalosporins (J01DB)**					
Cefazolin	162	30,775	8401	15,971,561	10.01 (8.56–11.7)
Cefalexin	150	30,787	15,184	15,964,778	5.12 (4.36–6.02)
Cefadroxil	12	30,925	1293	15,978,669	4.80 (2.72–8.47)
**Second-generation cephalosporins (J01DC)**					
Cefuroxime	311	30,626	11,920	15,968,042	13.60 (12.15–15.23)
Cefaclor	34	30,903	1157	15,978,805	15.19 (10.80–21.37)
Cefprozil	11	30,926	609	15,979,353	9.33 (5.14–16.94)
Cefoxitin	10	30,927	954	15,979,008	5.42 (2.90–10.10)
Cefotetan	2	30,935	101	15,979,861	10.23 (2.52–41.47)
**Third-generation cephalosporins (J01DD)**					
Ceftriaxone	548	30,389	25,953	15,954,009	11.09 (10.18–12.07)
Ceftazidime	126	30,811	5422	15,974,540	12.05 (10.09–14.38)
Cefdinir	89	30,848	5739	15,974,223	8.03 (6.51–9.90)
Cefotaxime	64	30,873	3257	15,976,705	10.17 (7.94–13.03)
Cefixime	50	30,887	1972	15,977,990	13.12 (9.90–17.37)
Cefpodoxime	26	30,911	698	15,979,264	19.26 (13.02–28.49)
Ceftazidime and beta-lactamase inhibitor	1	30,936	126	15,979,836	4.10 (0.57–29.33)
**Fourth-generation cephalosporins (J01DE)**					
Cefepime	288	30,649	10,696	15,969,266	14.03 (12.47–15.78)
**Monobactams (J01DF)**					
Aztreonam	42	30,895	6063	15,973,899	3.58 (2.64–4.85)
**Carbapenems (J01DH)**					
Meropenem	507	30,430	20,575	15,959,387	12.92 (11.83–14.12)
Ertapenem	166	30,771	5163	15,974,799	16.69 (14.30–19.49)
Imipenem and cilastatin	88	30,849	3343	15,976,619	13.63 (11.03–16.85)
**Other cephalosporins and penems (J01DI)**					
Ceftolozane and beta-lactamase inhibitor	7	30,930	749	15,979,213	4.83 (2.29–10.16)
Ceftaroline fosamil	5	30,932	501	15,979,461	5.16 (2.14–12.44)
Cefiderocol	1	30,936	112	15,979,850	4.61 (0.64–33.03)
**Trimethoprim and derivatives (J01EA)**					
Trimethoprim	165	30,772	8967	15,970,995	9.55 (8.18–11.14)
**Intermediate-acting sulfonamides (J01EC)**					
Sulfadiazine	4	30,933	1252	15,978,710	1.65 (0.62–4.40)
**Combinations of sulfonamides and trimethoprim, incl. derivatives (J01EE)**					
Sulfamethoxazole and trimethoprim	470	30,467	64,143	15,915,819	3.83 (3.49–4.19)
**Macrolides (J01FA)**					
Clarithromycin	300	30,637	26,676	15,953,286	5.86 (5.22–6.57)
Azithromycin	178	30,759	38,046	15,941,916	2.42 (2.09–2.81)
Erythromycin	118	30,819	14,595	15,965,367	4.19 (3.49–5.02)
**Lincosamides (J01FF)**					
Clindamycin	823	30,114	26,769	15,953,193	16.29 (15.18–17.47)
Lincomycin	7	30,930	230	15,979,732	15.72 (7.41–33.36)
**Streptogramins (J01FG)**					
Quinupristin/dalfopristin	2	30,935	102	15,979,860	10.13 (2.50–41.05)
**Streptomycins (J01GA)**					
Streptomycin	4	30,933	1002	15,978,960	2.06 (0.77–5.51)
**Other aminoglycosides (J01GB)**					
Gentamicin	210	30,727	12,309	15,967,653	8.87 (7.73–10.17)
Amikacin	142	30,795	11,578	15,968,384	6.36 (5.39–7.51)
Tobramycin	68	30,869	19,561	15,960,401	1.80 (1.42–2.28)
**Fluoroquinolones (J01MA)**					
Ciprofloxacin	1176	29,761	77,260	15,902,702	8.13 (7.67–8.63)
Levofloxacin	536	30,401	44,317	15,935,645	6.34 (5.82–6.91)
Moxifloxacin	75	30,862	11,887	15,968,075	3.26 (2.60–4.10)
Ofloxacin	39	30,898	5249	15,974,713	3.84 (2.80–5.26)
Gatifloxacin	33	30,904	1570	15,978,392	10.87 (7.70–15.34)
Delafloxacin	1	30,936	218	15,979,744	2.37 (0.33–16.9)
**Glycopeptide antibacterials (J01XA)**					
Vancomycin	1793	29,144	46,032	15,933,930	21.30 (20.29–22.36)
Dalbavancin	2	30,935	556	15,979,406	1.86 (0.46–7.45)
Telavancin	1	30,936	116	15,979,846	4.45 (0.62–31.88)
Oritavancin	1	30,936	791	15,979,171	0.65 (0.09–4.64)
**Polymyxins (J01XB)**					
Polymyxin B	14	30,923	975	15,978,987	7.42 (4.38–12.58)
Colistin	9	30,928	958	15,979,004	4.85 (2.52–9.36)
**Imidazole derivatives (J01XD)**					
Metronidazole	2004	28,933	49,926	15,930,036	22.10 (21.10–23.14)
Tinidazole	6	30,931	355	15,979,607	8.73 (3.90–19.57)
**Nitrofuran derivatives (J01XE)**					
Nitrofurantoin	15	30,922	2636	15,977,326	2.94 (1.77–4.88)
**Other antibacterials (J01XX)**					
Linezolid	168	30,769	20,272	15,959,690	4.30 (3.69–5.01)
Daptomycin	70	30,867	11,035	15,968,927	3.28 (2.59–4.15)
Fosfomycin	9	30,928	634	15,979,328	7.33 (3.80–14.16)
Tedizolid	3	30,934	499	15,979,463	3.11 (1.00–9.66)

Note: The classification of antibacterial agents is based on the Anatomical Therapeutic Chemical classification system, and the bold represents the drug category and its code. Abbreviations: AE, adverse event; *CI*, confidence interval; *ROR*, reporting odd ratio.

**Table 3:** Adjusted signal detection results for metronidazole at SMQ level and PT level.

Target PT	Drug of Interest with AE of Interest (*a*)	Other Drugs with AE of Interest (*b*)	Drug of Interest with Other AEs (*c*)	Other Drugs with Other AEs (*d*)	*ROR* (95% *CI*)
Antibiotic associated colitis	3	20	51,927	15,958,949	46.10 (13.70–155.14)
Clostridium bacteraemia	10	131	51,920	15,958,838	23.46 (12.33–44.64)
Clostridium colitis	49	750	51,881	15,958,219	20.10 (15.05–26.83)
Clostridium difficile colitis	535	7710	51,395	15,951,259	21.54 (19.72–23.52)
Clostridium difficile infection	725	15,071	51,205	15,943,898	14.98 (13.90–16.15)
Clostridial infection	140	2877	51,790	15,956,092	14.99 (12.65–17.77)
Clostridial sepsis	1	141	51,929	15,958,828	2.18 (0.30–15.58)
Clostridium test positive	104	1296	51,826	15,957,673	24.71 (20.23–30.18)
Gastroenteritis clostridial	4	277	51,926	15,958,692	4.44 (1.65–11.91)
Pseudomembranous colitis	140	1778	51,790	15,957,191	24.26 (20.42–28.82)
SMQ level	1863	29,074	50,067	15,929,895	20.39 (19.44–21.38)

Abbreviations: AE, adverse event; *CI*, confidence interval; PT, Preferred Term; *ROR*, reporting odd ratio.

**Table 4:** Adjusted signal detection results for vancomycin at SMQ level and PT level.

Target PT	Drug of Interest with AE of Interest (*a*)	Other Drugs with AE of Interest (*b*)	Drug of Interest with Other AEs (*c*)	Other Drugs with Other AEs (*d*)	*ROR* (95% *CI*)
Clostridium bacteraemia	10	131	47,815	15,962,943	25.48 (13.40–48.48)
Clostridium colitis	47	752	47,778	15,962,322	20.88 (15.55–28.04)
Clostridium difficile colitis	434	7811	47,391	15,955,263	18.71 (16.98–20.61)
Clostridium difficile infection	759	15,037	47,066	15,948,037	17.10 (15.89–18.41)
Clostridial infection	98	2919	47,727	15,960,155	11.23 (9.18–13.73)
Clostridial sepsis	8	134	47,817	15,962,940	19.93 (9.77–40.68)
Clostridium test positive	100	1300	47,725	15,961,774	25.73 (20.99–31.54)
Gastroenteritis clostridial	9	272	47,816	15,962,802	11.05 (5.69–21.46)
Pseudomembranous colitis	136	1782	47,689	15,961,292	25.54 (21.45–30.42)
SMQ level	1503	29,434	46,322	15,933,640	17.56 (16.66–18.51)

Note: There was no report of target drug-AE combination (*a* = 0) in antibiotic associated colitis (PT). Abbreviations: AE, adverse event; *CI*, confidence interval; PT, Preferred Term; *ROR*, reporting odd ratio.

**Table 5:** Two-by-two contingency tables for disproportionality analysis.

	Drug of Interest	Other Drugs	Total
AE of interest	*a*	*b*	*a* + *b*
Other AEs	*c*	*d*	*c* + *d*
Total	*a* + *c*	*b* + *d*	*a* + *b* + *c* + *d*

Abbreviations: AE, adverse event.

## Data Availability

Data are available on the FAERS database.

## Supplementary Materials

The following supporting information can be downloaded at: https://www.mdpi.com/article/10.3390/antibiotics12071109/s1, Table S1: Pharmacovigilance signal detection results for antibiotic associated colitis; Table S2: Pharmacovigilance signal detection results for clostridium bacteremia; Table S3: Pharmacovigilance signal detection results for clostridium colitis; Table S4: Pharmacovigilance signal detection results for clostridium difficile colitis; Table S5: Pharmacovigilance signal detection results for clostridium difficile infection; Table S6: Pharmacovigilance signal detection results for clostridial infection; Table S7: Pharmacovigilance signal detection results for clostridial sepsis; Table S8: Pharmacovigilance signal detection results for clostridium test positive; Table S9: Pharmacovigilance signal detection results for gastroenteritis clostridial; Table S10: Pharmacovigilance signal detection results for pseudomembranous colitis.
