# Supplementary material for: Real-World Data in Pharmacovigilance Database Provides a New Perspective for Understanding the Risk of Clostridium difficile Infection Associated with Antibacterial Drug Exposure
Source: Antibiotics (Basel). 2023 Jun 27;12(7):1109. doi: 10.3390/antibiotics12071109 (PMC10376141; doi:10.3390/antibiotics12071109)
Supplement: Supplementary file 1 [file antibiotics-12-01109-s001.zip › antibiotics-2441883-supplementary.pdf]

Table S1. Pharmacovigilance signal detection results for antibiotic associated colitis.

Medication	Drug of interest with AE of interest (a)	Other drugs with AE of interest (b)	Drug of interest with Other AEs (c)	Other drugs with Other AEs (d)	ROR (95% CI)
Tigecycline	1	22	4270	16,006,606	170.39 (22.96–1264.43)
Amoxicillin	2	21	57,923	15,952,953	26.23 (6.15–111.87)
Ceftriaxone	4	19	26,497	15,984,379	127.00 (43.20–373.34)
Meropenem	2	21	21,080	15,989,796	72.24 (16.94–308.12)
Sulfamethoxazole and trimethoprim	1	22	64,612	15,946,264	11.22 (1.51–83.23)
Amikacin	2	21	11,718	15,999,158	130.03 (30.49–554.65)
Levofloxacin	3	20	44,850	15,966,026	53.40 (15.87–179.71)
Moxifloxacin	1	22	11,961	15,998,915	60.80 (8.19–451.11)
Vancomycin	1	22	47,824	15,963,052	15.17 (2.04–112.57)
Metronidazole	3	20	51,927	15,958,949	46.10 (13.7–155.14)
Linezolid	1	22	20,439	15,990,437	35.56 (4.79–263.84)

Note: Signal detection results for no report of target drug-AE combination (a = 0) is omitted.
Abbreviations: AE, adverse event; CI, confidence interval; ROR, reporting odd ratio.

Table S2. Pharmacovigilance signal detection results for clostridium bacteraemia.

Medication	Drug of interest with AE of interest (a)	Other drugs with AE of interest (b)	Drug of interest with Other AEs (c)	Other drugs with Other AEs (d)	ROR (95% CI)
Doxycycline	1	140	46,926	15,963,832	2.43 (0.34–17.37)
Tigecycline	1	140	4270	16,006,488	26.78 (3.74–191.47)
Ampicillin	1	140	6276	16,004,482	18.22 (2.55–130.24)
Amoxicillin	3	138	57,922	15,952,836	5.99 (1.91–18.79)
Ampicillin and beta-lactamase inhibitor	1	140	2179	16,008,579	52.48 (7.34–375.33)
Amoxicillin and beta-lactamase inhibitor	1	140	22,159	15,988,599	5.15 (0.72–36.85)
Piperacillin and beta-lactamase inhibitor	3	138	19,785	15,990,973	17.57 (5.60–55.15)
Cefazolin	2	139	8561	16,002,197	26.89 (6.66–108.63)
Cefuroxime	2	139	12,229	15,998,529	18.82 (4.66–76.03)
Cefotaxime	4	137	3317	16,007,441	140.90 (52.11–381.02)
Ceftazidime	3	138	5545	16,005,213	62.75 (19.99–197.01)
Ceftriaxone	5	136	26,496	15,984,262	22.18 (9.08–54.15)
Meropenem	5	136	21,077	15,989,681	27.89 (11.42–68.10)
Trimethoprim	1	140	9131	16,001,627	12.52 (1.75–89.50)
Sulfamethoxazole and trimethoprim	3	138	64610	15,946,148	5.37 (1.71–16.84)
Clindamycin	1	140	27,591	15,983,167	4.14 (0.58–29.58)
Tobramycin	1	140	19,628	15,991,130	5.82 (0.81–41.61)
Gentamicin	2	139	12517	15,998,241	18.39 (4.55–74.28)
Amikacin	3	138	11,717	15,999,041	29.68 (9.46–93.18)
Ofloxacin	1	140	5287	16,005,471	21.62 (3.02–154.62)
Ciprofloxacin	8	133	78,428	15,932,330	12.22 (5.99–24.94)
Levofloxacin	2	139	44,851	15,965,907	5.12 (1.27–20.69)
Vancomycin	10	131	47,815	15,962,943	25.48 (13.40–48.48)
Metronidazole	10	131	51,920	15,958,838	23.46 (12.33–44.64)
Daptomycin	1	140	11,104	15,999,654	10.29 (1.44–73.59)

Note: Signal detection results for no report of target drug-AE combination (a = 0) is omitted.
Abbreviations: AE, adverse event; CI, confidence interval; ROR, reporting odd ratio.

Table S3. Pharmacovigilance signal detection results for clostridium colitis.

Medication	Drug of interest with AE of interest (a)	Other drugs with AE of interest (b)	Drug of interest with Other AEs (c)	Other drugs with Other AEs (d)	ROR (95% CI)
Doxycycline	5	794	46,922	15,963,178	2.14 (0.89–5.16)
Minocycline	3	796	12,085	15,998,015	4.99 (1.61–15.50)
Ampicillin	2	797	6275	16,003,825	6.40 (1.60–25.64)
Amoxicillin	26	773	57,899	15,952,201	9.27 (6.27–13.70)
Ampicillin and beta-lactamase inhibitor	7	792	2173	16,007,927	65.11 (30.90–137.19)
Amoxicillin and beta-lactamase inhibitor	25	774	22135	15,987,965	23.33 (15.66–34.75)
Piperacillin and beta-lactamase inhibitor	13	786	19,775	15,990,325	13.37 (7.73–23.14)
Cefalexin	3	796	15,331	15,994,769	3.93 (1.27–12.22)
Cefazolin	9	790	8554	16,001,546	21.31 (11.04–41.13)
Cefadroxil	3	796	1302	16,008,798	46.34 (14.89–144.18)
Cefuroxime	8	791	12,223	15,997,877	13.24 (6.60–26.57)
Cefotaxime	12	787	3309	16,006,791	73.76 (41.67–130.57)
Ceftazidime	7	792	5541	16,004,559	25.53 (12.12–53.75)
Ceftriaxone	19	780	26,482	15,983,618	14.7 (9.33–23.18)
Cefixime	2	797	2020	16,008,080	19.89 (4.96–79.71)
Cefdinir	1	798	5827	16,004,273	3.44 (0.48–24.47)
Cefepime	3	796	10,981	15,999,119	5.49 (1.77–17.06)
Aztreonam	1	798	6104	16,003,996	3.29 (0.46–23.36)
Meropenem	17	782	21,065	15,989,035	16.50 (10.20–26.69)
Ertapenem	4	795	5325	16,004,775	15.12 (5.66–40.41)
Imipenem and cilastatin	10	789	3421	16,006,679	59.30 (31.75–110.75)
Trimethoprim	10	789	9122	16,000,978	22.23 (11.91–41.50)
Sulfamethoxazole and trimethoprim	22	777	64,591	15,945,509	6.99 (4.58–10.68)
Erythromycin	12	787	14,701	15,995,399	16.59 (9.38–29.35)
Clarithromycin	20	779	26,956	15,983,144	15.22 (9.76–23.73)
Azithromycin	6	793	38,218	15,971,882	3.16 (1.42–7.06)
Clindamycin	27	772	27,565	15,982,535	20.28 (13.81–29.77)
Tobramycin	2	797	19,627	15,990,473	2.04 (0.51–8.19)
Gentamicin	15	784	12,504	15,997,596	24.48 (14.68–40.81)
Amikacin	8	791	11,712	15,998,388	13.82 (6.88–27.73)
Ofloxacin	2	797	5286	16,004,814	7.60 (1.90–30.44)

Ciprofloxacin	45	754	78,391	15,931,709	12.13 (8.98–16.39)
Levofloxacin	24	775	44,829	15,965,271	11.03 (7.35–16.56)
Moxifloxacin	2	797	11,960	15,998,140	3.36 (0.84–13.45)
Gatifloxacin	21	778	1582	16,008,518	273.14 (176.58–422.51)
Vancomycin	59	740	47,766	15,962,334	26.64 (20.44–34.74)
Polymyxin B	1	798	988	16,009,112	20.31 (2.85–144.47)
Metronidazole	74	725	51,856	15,958,244	31.41 (24.72–39.90)
Nitrofurantoin	1	798	2650	16,007,450	7.57 (1.06–53.83)
Fosfomycin	1	798	642	16,009,458	31.25 (4.39–222.46)
Linezolid	2	797	20,438	15,989,662	1.96 (0.49–7.86)

Note: Signal detection results for no report of target drug-AE combination (a = 0) is omitted.
Abbreviations: AE, adverse event; CI, confidence interval; ROR, reporting odd ratio.

Table S4. Pharmacovigilance signal detection results for clostridium difficile colitis.

Medication	Drug of interest with AE of interest (a)	Other drugs with AE of interest (b)	Drug of interest with Other AEs (c)	Other drugs with Other AEs (d)	ROR (95% CI)
Doxycycline	58	8187	46,869	15,955,785	2.41 (1.86–3.12)
Tetracycline	1	8244	372	16,002,282	5.22 (0.73–37.15)
Minocycline	12	8233	12,076	15,990,578	1.93 (1.10–3.40)
Tigecycline	40	8205	4231	15,998,423	18.43 (13.49–25.19)
Combinations of tetracyclines	8	8237	825	16,001,829	18.84 (9.39–37.81)
Ampicillin	30	8215	6247	15,996,407	9.35 (6.53–13.39)
Amoxicillin	161	8084	57,764	15,944,890	5.50 (4.70–6.43)
Benzylpenicillin	4	8241	1617	16,001,037	4.80 (1.80–12.82)
Phenoxymethylpenicillin	2	8243	1600	16,001,054	2.43 (0.61–9.71)
Oxacillin	4	8241	899	16,001,755	8.64 (3.23–23.08)
Nafcillin	7	8238	805	16,001,849	16.89 (8.02–35.56)
Ampicillin and beta-lactamase inhibitor	17	8228	2163	16,000,491	15.28 (9.48–24.64)
Amoxicillin and beta-lactamase inhibitor	135	8110	22,025	15,980,629	12.08 (10.18–14.32)
Piperacillin and beta-lactamase inhibitor	150	8095	19,638	15,983,016	15.08 (12.82–17.74)
Cefalexin	53	8192	15,281	15,987,373	6.77 (5.16–8.87)
Cefazolin	60	8185	8503	15,994,151	13.79 (10.69–17.79)
Cefadroxil	3	8242	1302	16,001,352	4.47 (1.44–13.89)
Cefoxitin	3	8242	961	16,001,693	6.06 (1.95–18.83)
Cefuroxime	92	8153	12,139	15,990,515	14.86 (12.09–18.27)
Cefaclor	2	8243	1189	16,001,465	3.27 (0.82–13.07)
Cefotetan	1	8244	102	16,002,552	19.03 (2.65–136.42)
Cefprozil	4	8241	616	16,002,038	12.61 (4.72–33.71)
Cefotaxime	28	8217	3293	15,999,361	16.56 (11.41–24.03)
Ceftazidime	46	8199	5502	15,997,152	16.31 (12.19–21.82)
Ceftriaxone	169	8076	26,332	15,976,322	12.70 (10.90–14.79)
Cefixime	17	8228	2005	16,000,649	16.49 (10.22–26.59)
Cefpodoxime	12	8233	712	16,001,942	32.76 (18.51–57.98)
Cefdinir	25	8220	5803	15,996,851	8.38 (5.66–12.43)
Ceftazidime and beta-lactamase inhibitor	1	8244	126	16,002,528	15.41 (2.15–110.24)
Cefepime	121	8124	10,863	15,991,791	21.93 (18.31–26.26)

Aztreonam	14	8231	6091	15,996,563	4.47 (2.64–7.55)
Meropenem	183	8062	20,899	15,981,755	17.36 (14.98–20.11)
Ertapenem	44	8201	5285	15,997,369	16.24 (12.06–21.87)
Imipenem and cilastatin	28	8217	3403	15,999,251	16.02 (11.04–23.25)
Ceftaroline fosamil	1	8244	505	16,002,149	3.84 (0.54–27.34)
Cefiderocol	1	8244	112	16,002,542	17.33 (2.42–124.13)
Ceftolozane and beta-lactamase inhibitor	1	8244	755	16,001,899	2.57 (0.36–18.28)
Trimethoprim	19	8226	9113	15,993,541	4.05 (2.58–6.36)
Sulfamethoxazole and trimethoprim	170	8075	64,443	15,938,211	5.21 (4.47–6.06)
Erythromycin	30	8215	14,683	15,987,971	3.98 (2.78–5.69)
Clarithromycin	65	8180	26,911	15,975,743	4.72 (3.69–6.02)
Azithromycin	78	8167	38,146	15,964,508	4 (3.20–5.00)
Clindamycin	211	8034	27,381	15,975,273	15.32 (13.36–17.58)
Lincomycin	1	8244	236	16,002,418	8.22 (1.15–58.64)
Quinupristin/dalfopristin	1	8244	103	16,002,551	18.85 (2.63–135.08)
Streptomycin	2	8243	1004	16,001,650	3.87 (0.97–15.49)
Tobramycin	22	8223	19,607	15,983,047	2.18 (1.43–3.31)
Gentamicin	44	8201	12,475	15,990,179	6.88 (5.11–9.25)
Amikacin	64	8181	11,656	15,990,998	10.73 (8.39–13.73)
Ofloxacin	10	8235	5278	15,997,376	3.68 (1.98–6.85)
Ciprofloxacin	416	7829	78,020	15,924,634	10.85 (9.82–11.97)
Levofloxacin	169	8076	44,684	15,957,970	7.47 (6.42–8.71)
Moxifloxacin	30	8215	11,932	15,990,722	4.89 (3.42–7.01)
Gatifloxacin	5	8240	1598	16,001,056	6.08 (2.52–14.62)
Delafloxacin	1	8244	218	16,002,436	8.90 (1.25–63.51)
Vancomycin	509	7736	47,316	15,955,338	22.19 (20.27–24.28)
Dalbavancin	2	8243	556	16,002,098	6.98 (1.74–28.00)
Colistin	7	8238	960	16,001,694	14.16 (6.73–29.8)
Polymyxin B	6	8239	983	16,001,671	11.85 (5.31–26.46)
Metronidazole	620	7625	51,310	15,951,344	25.28 (23.28–27.45)
Tinidazole	2	8243	359	16,002,295	10.82 (2.69–43.42)
Nitrofurantoin	3	8242	2648	16,000,006	2.20 (0.71–6.83)
Fosfomycin	4	8241	639	16,002,015	12.15 (4.55–32.49)
Linezolid	78	8167	20,362	15,982,292	7.50 (6.00–9.37)
Daptomycin	27	8218	11,078	15,991,576	4.74 (3.25–6.92)
Tedizolid	1	8244	501	16,002,153	3.87 (0.54–27.56)

Note: Signal detection results for no report of target drug-AE combination (a = 0) is omitted.
Abbreviations: AE, adverse event; CI, confidence interval; ROR, reporting odd ratio.

Table S5. Pharmacovigilance signal detection results for clostridium difficile infection.

Medication	Drug of interest with AE of interest (a)	Other drugs with AE of interest (b)	Drug of interest with Other AEs (c)	Other drugs with Other AEs (d)	ROR (95% CI)
Doxycycline	141	15655	46,786	15,948,317	3.07 (2.60–3.62)
Tetracycline	1	15795	372	15,994,731	2.72 (0.38–19.38)
Minocycline	10	15786	12,078	15,983,025	0.84 (0.45–1.56)
Tigecycline	22	15774	4249	15,990,854	5.25 (3.45–7.98)
Combinations of tetracyclines	8	15788	825	15,994,278	9.82 (4.90–19.71)
Ampicillin	22	15774	6255	15,988,848	3.57 (2.35–5.42)
Amoxicillin	277	15519	57,648	15,937,455	4.93 (4.38–5.56)
Benzylpenicillin	2	15794	1619	15,993,484	1.25 (0.31–5.01)
Phenoxymethylpenicillin	7	15789	1595	15,993,508	4.45 (2.12–9.34)
Dicloxacillin	1	15795	116	15,994,987	8.73 (1.22–62.5)
Oxacillin	6	15790	897	15,994,206	6.78 (3.04–15.12)
Nafcillin	1	15795	811	15,994,292	1.25 (0.18–8.88)
Ampicillin and beta-lactamase inhibitor	5	15791	2175	15,992,928	2.33 (0.97–5.60)
Amoxicillin and beta-lactamase inhibitor	113	15683	22,047	15,973,056	5.22 (4.34–6.28)
Piperacillin and beta-lactamase inhibitor	160	15636	19,628	15,975,475	8.33 (7.12–9.74)
Cefalexin	61	15735	15,273	15,979,830	4.06 (3.15–5.22)
Cefazolin	49	15747	8514	15,986,589	5.84 (4.41–7.74)
Cefadroxil	6	15790	1299	15,993,804	4.68 (2.10–10.43)
Cefoxitin	2	15794	962	15,994,141	2.11 (0.53–8.43)
Cefuroxime	133	15663	12,098	15,983,005	11.22 (9.45–13.32)
Cefaclor	25	15771	1166	15,993,937	21.74 (14.63–32.32)
Cefotetan	1	15795	102	15,995,001	9.93 (1.39–71.17)
Cefprozil	7	15789	613	15,994,490	11.57 (5.49–24.37)
Cefotaxime	12	15784	3309	15,991,794	3.67 (2.08–6.48)
Ceftazidime	37	15759	5511	15,989,592	6.81 (4.93–9.42)
Ceftriaxone	192	15604	26,309	15,968,794	7.47 (6.47–8.62)
Cefixime	17	15779	2005	15,993,098	8.59 (5.33–13.86)
Cefpodoxime	4	15792	720	15,994,383	5.63 (2.11–15.03)
Cefdinir	45	15751	5783	15,989,320	7.90 (5.89–10.60)
Cefepime	98	15698	10,886	15,984,217	9.17 (7.51–11.19)
Aztreonam	17	15779	6088	15,989,015	2.83 (1.76–4.56)

Meropenem	180	15616	20,902	15,974,201	8.81 (7.60–10.21)
Ertapenem	85	15711	5244	15,989,859	16.5 (13.31–20.45)
Imipenem and cilastatin	15	15781	3416	15,991,687	4.45 (2.68–7.39)
Ceftaroline fosamil	3	15793	503	15,994,600	6.04 (1.94–18.79)
Ceftolozane and beta-lactamase inhibitor	5	15791	751	15,994,352	6.74 (2.80–16.25)
Trimethoprim	98	15698	9034	15,986,069	11.05 (9.05–13.49)
Sulfadiazine	4	15792	1252	15,993,851	3.24 (1.21–8.64)
Sulfamethoxazole and trimethoprim	184	15612	64,429	15,930,674	2.91 (2.52–3.37)
Erythromycin	39	15757	14,674	15,980,429	2.7 (1.97–3.69)
Clarithromycin	131	15665	26,845	15,968,258	4.97 (4.19–5.91)
Azithromycin	44	15752	38,180	15,956,923	1.17 (0.87–1.57)
Clindamycin	398	15398	27,194	15,967,909	15.18 (13.73–16.78)
Lincomycin	3	15793	234	15,994,869	12.98 (4.16–40.56)
Quinupristin/dalfopristin	1	15795	103	15,995,000	9.83 (1.37–70.47)
Tobramycin	26	15770	19,603	15,975,500	1.34 (0.91–1.97)
Gentamicin	96	15700	12,423	15,982,680	7.87 (6.43–9.62)
Amikacin	30	15766	11,690	15,983,413	2.60 (1.82–3.72)
Ofloxacin	19	15777	5269	15,989,834	3.65 (2.33–5.74)
Ciprofloxacin	506	15290	77,930	15,917,173	6.76 (6.18–7.39)
Levofloxacin	213	15583	44,640	15,950,463	4.88 (4.26–5.59)
Moxifloxacin	26	15770	11,936	15,983,167	2.21 (1.50–3.24)
Vancomycin	943	14853	46,882	15,948,221	21.60 (20.21–23.08)
Telavancin	1	15795	116	15,994,987	8.73 (1.22–62.50)
Dalbavancin	2	15794	556	15,994,547	3.64 (0.91–14.60)
Oritavancin	1	15795	791	15,994,312	1.28 (0.18–9.10)
Metronidazole	939	14857	50,991	15,944,112	19.76 (18.49–21.12)
Tinidazole	2	15794	359	15,994,744	5.64 (1.41–22.65)
Nitrofurantoin	5	15791	2646	15,992,457	1.91 (0.8–4.60)
Fosfomycin	3	15793	640	15,994,463	4.75 (1.53–14.76)
Linezolid	36	15760	20,404	15,974,699	1.79 (1.29–2.48)
Daptomycin	20	15776	11,085	15,984,018	1.83 (1.18–2.84)
Tedizolid	1	15795	501	15,994,602	2.02 (0.28–14.38)

Note: Signal detection results for no report of target drug-AE combination (a = 0) is omitted.
Abbreviations: AE, adverse event; CI, confidence interval; ROR, reporting odd ratio.

Table S6. Pharmacovigilance signal detection results for clostridial infection.

Medication	Drug of interest with AE of interest (a)	Other drugs with AE of interest (b)	Drug of interest with Other AEs (c)	Other drugs with Other AEs (d)	ROR (95% CI)
Doxycycline	16	3001	46,911	15,960,971	1.81 (1.11–2.97)
Minocycline	2	3015	12,086	15,995,796	0.88 (0.22–3.51)
Tigecycline	8	3009	4263	16,003,619	9.98 (4.98–19.99)
Combinations of tetracyclines	2	3015	831	16,007,051	12.78 (3.19–51.2)
Ampicillin	14	3003	6263	16,001,619	11.91 (7.04–20.15)
Amoxicillin	31	2986	57,894	15,949,988	2.86 (2.01–4.07)
Benzylpenicillin	1	3016	1620	16,006,262	3.28 (0.46–23.28)
Oxacillin	1	3016	902	16,006,980	5.88 (0.83–41.83)
Nafcillin	1	3016	811	16,007,071	6.54 (0.92–46.53)
Ampicillin and beta-lactamase inhibitor	7	3010	2173	16,005,709	17.13 (8.15–36.01)
Amoxicillin and beta-lactamase inhibitor	24	2993	22,136	15,985,746	5.79 (3.87–8.66)
Piperacillin and beta-lactamase inhibitor	55	2962	19,733	15,988,149	15.04 (11.52–19.65)
Cefalexin	18	2999	15,316	15,992,566	6.27 (3.94–9.96)
Cefazolin	14	3003	8549	15,999,333	8.72 (5.16–14.76)
Cefoxitin	1	3016	963	16,006,919	5.51 (0.78–39.18)
Cefuroxime	26	2991	12,205	15,995,677	11.39 (7.74–16.77)
Cefaclor	1	3016	1190	16,006,692	4.46 (0.63–31.7)
Cefotaxime	5	3012	3316	16,004,566	8.01 (3.33–19.28)
Ceftazidime	12	3005	5536	16,002,346	11.54 (6.54–20.36)
Ceftriaxone	43	2974	26,458	15,981,424	8.73 (6.46–11.8)
Cefpodoxime	3	3014	721	16,007,161	22.1 (7.11–68.72)
Cefdinir	12	3005	5816	16,002,066	10.99 (6.23–19.38)
Cefepime	23	2994	10,961	15,996,921	11.21 (7.44–16.91)
Aztreonam	1	3016	6104	16,001,778	0.87 (0.12–6.17)
Meropenem	34	2983	21,048	15,986,834	8.66 (6.17–12.14)
Ertapenem	11	3006	5318	16,002,564	11.01 (6.09–19.92)
Imipenem and cilastatin	14	3003	3417	16,004,465	21.84 (12.9–36.95)
Ceftolozane and beta-lactamase inhibitor	1	3016	755	16,007,127	7.03 (0.99–49.99)
Trimethoprim	12	3005	9120	15,998,762	7.01 (3.97–12.35)
Sulfamethoxazole and	43	2974	64,570	15,943,312	3.57 (2.64–4.82)

trimethoprim					
Erythromycin	9	3008	14,704	15,993,178	3.25 (1.69–6.26)
Clarithromycin	16	3001	26,960	15,980,922	3.16 (1.93–5.17)
Azithromycin	26	2991	38,198	15,969,684	3.63 (2.47–5.35)
Clindamycin	76	2941	27,516	15,980,366	15.01 (11.95–18.85)
Tobramycin	4	3013	19,625	15,988,257	1.08 (0.41–2.88)
Gentamicin	22	2995	12,497	15,995,385	9.40 (6.18–14.31)
Amikacin	17	3000	11,703	15,996,179	7.75 (4.81–12.48)
Ciprofloxacin	89	2928	78,347	15,929,535	6.18 (5.00–7.63)
Levofloxacin	55	2962	44,798	15,963,084	6.62 (5.07–8.64)
Moxifloxacin	6	3011	11,956	15,995,926	2.67 (1.20–5.94)
Gatifloxacin	7	3010	1596	16,006,286	23.32 (11.09–49.05)
Vancomycin	108	2909	47,717	15,960,165	12.42 (10.25–15.05)
Colistin	2	3015	965	16,006,917	11.00 (2.75–44.08)
Polymyxin B	2	3015	987	16,006,895	10.76 (2.69–43.1)
Metronidazole	171	2846	51,759	15,956,123	18.52 (15.87–21.62)
Nitrofurantoin	4	3013	2647	16,005,235	8.03 (3.01–21.42)
Linezolid	21	2996	20,419	15,987,463	5.49 (3.57–8.43)
Daptomycin	8	3009	11,097	15,996,785	3.83 (1.91–7.67)

Note: Signal detection results for no report of target drug-AE combination (a = 0) is omitted.
Abbreviations: AE, adverse event; CI, confidence interval; ROR, reporting odd ratio.

Table S7. Pharmacovigilance signal detection results for clostridial sepsis.

Medication	Drug of interest with AE of interest (a)	Other drugs with AE of interest (b)	Drug of interest with Other AEs (c)	Other drugs with Other AEs (d)	ROR (95% CI)
Doxycycline	1	141	46,926	15,963,831	2.41 (0.34–17.25)
Amoxicillin	1	141	57,924	15,952,833	1.95 (0.27–13.96)
Oxacillin	1	141	902	16,009,855	125.88 (17.59–900.87)
Piperacillin and beta-lactamase inhibitor	4	138	19,784	15,990,973	23.43 (8.67–63.32)
Cefazolin	1	141	8562	16,002,195	13.26 (1.85–94.77)
Ceftazidime	2	140	5546	16,005,211	41.23 (10.21–166.52)
Ceftriaxone	6	136	26,495	15,984,262	26.62 (11.75–60.29)
Cefdinir	1	141	5827	16,004,930	19.48 (2.72–139.28)
Meropenem	9	133	21,073	15,989,684	51.35 (26.14–100.87)
Ertapenem	2	140	5327	16,005,430	42.92 (10.63–173.37)
Trimethoprim	3	139	9129	16,001,628	37.83 (12.05–118.76)
Clindamycin	4	138	27,588	15,983,169	16.79 (6.21–45.38)
Lincomycin	1	141	236	16,010,521	481.14 (67.03–3453.82)
Amikacin	1	141	11,719	15,999,038	9.68 (1.35–69.22)
Ciprofloxacin	4	138	78,432	15,932,325	5.89 (2.18–15.91)
Levofloxacin	6	136	44,847	15,965,910	15.71 (6.93–35.58)
Vancomycin	9	133	47,816	15,962,941	22.59 (11.50–44.38)
Metronidazole	1	141	51,929	15,958,828	2.18 (0.30–15.58)
Nitrofurantoin	1	141	2650	16,008,107	42.84 (5.99–306.38)
Linezolid	5	137	20,435	15,990,322	28.56 (11.7–69.72)
Daptomycin	1	141	11,104	15,999,653	10.22 (1.43–73.06)

Note: Signal detection results for no report of target drug-AE combination (a = 0) is omitted.
Abbreviations: AE, adverse event; CI, confidence interval; ROR, reporting odd ratio.

Table S8. Pharmacovigilance signal detection results for clostridium test positive.

Medication	Drug of interest with AE of interest (a)	Other drugs with AE of interest (b)	Drug of interest with Other AEs (c)	Other drugs with Other AEs (d)	ROR (95% CI)
Doxycycline	3	1397	46,924	15,962,575	0.73 (0.24–2.27)
Tigecycline	2	1398	4269	16,005,230	5.36 (1.34–21.47)
Ampicillin	3	1397	6274	16,003,225	5.48 (1.76–17.01)
Amoxicillin	28	1372	57,897	15,951,602	5.62 (3.87–8.18)
Benzylpenicillin	2	1398	1619	16,007,880	14.15 (3.53–56.66)
Phenoxymethylpenicillin	1	1399	1601	16,007,898	7.15 (1.01–50.81)
Nafcillin	1	1399	811	16,008,688	14.11 (1.98–100.36)
Ampicillin and beta-lactamase inhibitor	9	1391	2171	16,007,328	47.71 (24.74–92.01)
Amoxicillin and beta-lactamase inhibitor	18	1382	22,142	15,987,357	9.4 (5.91–14.97)
Piperacillin and beta-lactamase inhibitor	63	1337	19,725	15,989,774	38.20 (29.66–49.20)
Cefalexin	14	1386	15,320	15,994,179	10.55 (6.23–17.86)
Cefazolin	12	1388	8551	16,000,948	16.18 (9.16–28.57)
Cefoxitin	1	1399	963	16,008,536	11.88 (1.67–84.50)
Cefuroxime	30	1370	12,201	15,997,298	28.71 (19.99–41.24)
Cefaclor	2	1398	1189	16,008,310	19.26 (4.81–77.18)
Cefotaxime	1	1399	3320	16,006,179	3.45 (0.48–24.49)
Ceftazidime	8	1392	5540	16,003,959	16.6 (8.28–33.28)
Ceftriaxone	68	1332	26,433	15,983,066	30.87 (24.19–39.4)
Cefixime	6	1394	2016	16,007,483	34.18 (15.31–76.29)
Cefpodoxime	1	1399	723	16,008,776	15.83 (2.22–112.59)
Cefdinir	5	1395	5823	16,003,676	9.85 (4.09–23.71)
Cefepime	25	1375	10,959	15,998,540	26.54 (17.86–39.44)
Aztreonam	4	1396	6101	16,003,398	7.52 (2.82–20.06)
Meropenem	50	1350	21,032	15,988,467	28.16 (21.22–37.35)
Ertapenem	31	1369	5298	16,004,201	68.4 (47.87–97.75)
Imipenem and cilastatin	12	1388	3419	16,006,080	40.47 (22.91–71.51)
Ceftaroline fosamil	1	1399	505	16,008,994	22.66 (3.18–161.29)
Trimethoprim	23	1377	9109	16,000,390	29.34 (19.42–44.32)
Sulfamethoxazole and trimethoprim	28	1372	64,585	15,944,914	5.04 (3.47–7.33)
Erythromycin	14	1386	14,699	15,994,800	10.99 (6.49–18.61)
Clarithromycin	19	1381	26,957	15,982,542	8.16 (5.19–12.83)

Azithromycin	8	1392	38,216	15,971,283	2.40 (1.20–4.81)
Clindamycin	56	1344	27,536	15,981,963	24.18 (18.51–31.60)
Streptomycin	1	1399	1005	16,008,494	11.39 (1.60–80.97)
Tobramycin	3	1397	19,626	15,989,873	1.75 (0.56–5.43)
Gentamicin	15	1385	12,504	15,996,995	13.86 (8.33–23.05)
Amikacin	11	1389	11,709	15,997,790	10.82 (5.98–19.59)
Ciprofloxacin	61	1339	78,375	15,931,124	9.26 (7.16–11.97)
Levofloxacin	24	1376	44,829	15,964,670	6.21 (4.15–9.30)
Moxifloxacin	5	1395	11,957	15,997,542	4.80 (1.99–11.54)
Vancomycin	109	1291	47,716	15,961,783	28.24 (23.22–34.35)
Colistin	2	1398	965	16,008,534	23.73 (5.92–95.13)
Polymyxin B	2	1398	987	16,008,512	23.20 (5.79–93.00)
Metronidazole	108	1292	51,822	15,957,677	25.74 (21.15–31.33)
Linezolid	18	1382	20,422	15,989,077	10.20 (6.40–16.24)
Daptomycin	5	1395	11,100	15,998,399	5.17 (2.15–12.43)

Note: Signal detection results for no report of target drug-AE combination (a = 0) is omitted.
Abbreviations: AE, adverse event; CI, confidence interval; ROR, reporting odd ratio.

Table S9. Pharmacovigilance signal detection results for gastroenteritis clostridial.

Medication	Drug of interest with AE of interest (a)	Other drugs with AE of interest (b)	Drug of interest with Other AEs (c)	Other drugs with Other AEs (d)	ROR (95% CI)
Doxycycline	1	280	46,926	15,963,692	1.21 (0.17–8.66)
Amoxicillin	8	273	57,917	15,952,701	8.07 (4.00–16.30)
Amoxicillin and beta-lactamase inhibitor	4	277	22,156	15,988,462	10.42 (3.88–27.96)
Piperacillin and beta-lactamase inhibitor	8	273	19,780	15,990,838	23.69 (11.73–47.86)
Cefuroxime	7	274	12,224	15,998,394	33.44 (15.79–70.81)
Cefaclor	1	280	1190	16,009,428	48.05 (6.74–342.58)
Ceftazidime	3	278	5545	16,005,073	31.15 (9.98–97.20)
Ceftriaxone	16	265	26,485	15,984,133	36.44 (22.00–60.36)
Cefepime	2	279	10,982	15,999,636	10.44 (2.60–41.97)
Meropenem	2	279	21,080	15,989,538	5.44 (1.35–21.85)
Ertapenem	1	280	5328	16,005,290	10.73 (1.51–76.45)
Imipenem and cilastatin	2	279	3429	16,007,189	33.46 (8.32–134.53)
Sulfamethoxazole and trimethoprim	2	279	64,611	15,946,007	1.77 (0.44–7.11)
Clarithromycin	7	274	26,969	15,983,649	15.14 (7.15–32.06)
Azithromycin	2	279	38,222	15,972,396	3 (0.75–12.04)
Clindamycin	3	278	27,589	15,983,029	6.25 (2.00–19.50)
Tobramycin	1	280	19,628	15,990,990	2.91 (0.41–20.73)
Amikacin	1	280	11,719	15,998,899	4.88 (0.68–34.74)
Ciprofloxacin	13	268	78,423	15,932,195	9.85 (5.65–17.20)
Levofloxacin	9	272	44,844	15,965,774	11.78 (6.06–22.89)
Moxifloxacin	1	280	11,961	15,998,657	4.78 (0.67–34.04)
Vancomycin	10	271	47,815	15,962,803	12.32 (6.55–23.16)
Metronidazole	9	272	51,921	15,958,697	10.17 (5.23–19.76)
Linezolid	2	279	20,438	15,990,180	5.61 (1.40–22.54)
Daptomycin	4	277	11,101	15,999,517	20.81 (7.75–55.86)

Note: Signal detection results for no report of target drug-AE combination (a = 0) is omitted.
Abbreviations: AE, adverse event; CI, confidence interval; ROR, reporting odd ratio.

Table S10. Pharmacovigilance signal detection results for pseudomembranous colitis.

Medication	Drug of interest with AE of interest (a)	Other drugs with AE of interest (b)	Drug of interest with Other AEs (c)	Other drugs with Other AEs (d)	ROR (95% CI)
Doxycycline	9	1909	46,918	15,962,063	1.6 (0.83–3.09)
Minocycline	7	1911	12,081	15,996,900	4.85 (2.31–10.19)
Tigecycline	9	1909	4262	16,004,719	17.7 (9.19–34.1)
Combinations of tetracyclines	2	1916	831	16,008,150	20.11 (5.02–80.6)
Chloramphenicol	1	1917	26	16,008,955	321.19 (43.56–2368.2)
Ampicillin	22	1896	6255	16,002,726	29.69 (19.49–45.23)
Amoxicillin	46	1872	57,879	15,951,102	6.77 (5.05–9.07)
Benzylpenicillin	1	1917	1620	16,007,361	5.15 (0.73–36.63)
Benzathine benzylpenicillin	1	1917	559	16,008,422	14.94 (2.10–106.3)
Oxacillin	2	1916	901	16,008,080	18.55 (4.63–74.32)
Ampicillin and beta-lactamase inhibitor	9	1909	2171	16,006,810	34.76 (18.03–67.00)
Amoxicillin and beta-lactamase inhibitor	24	1894	22,136	15,986,845	9.15 (6.12–13.69)
Piperacillin and beta-lactamase inhibitor	55	1863	19,733	15,989,248	23.92 (18.29–31.29)
Cefalexin	3	1915	15,331	15,993,650	1.63 (0.53–5.07)
Cefazolin	22	1896	8541	16,000,440	21.74 (14.27–33.11)
Cefoxitin	4	1914	960	16,008,021	34.85 (13.04–93.14)
Cefuroxime	48	1870	12,183	15,996,798	33.70 (25.29–44.91)
Cefaclor	4	1914	1187	16,007,794	28.18 (10.55–75.30)
Cefotetan	1	1917	102	16,008,879	81.87 (11.42–587.14)
Cefotaxime	7	1911	3314	16,005,667	17.69 (8.42–37.19)
Ceftazidime	22	1896	5526	16,003,455	33.60 (22.05–51.20)
Ceftriaxone	73	1845	26,428	15,982,553	23.93 (18.93–30.24)
Cefixime	9	1909	2013	16,006,968	37.49 (19.45–72.27)
Cefpodoxime	7	1911	717	16,008,264	81.78 (38.8–172.4)
Cefdinir	5	1913	5823	16,003,158	7.18 (2.99–17.28)
Cefepime	31	1887	10,953	15,998,028	24.00 (16.82–34.24)
Aztreonam	9	1909	6096	16,002,885	12.38 (6.43–23.83)
Meropenem	71	1847	21,011	15,987,970	29.25 (23.07–37.09)
Ertapenem	2	1916	5327	16,003,654	3.14 (0.78–12.55)
Imipenem and cilastatin	22	1896	3409	16,005,572	54.48 (35.74–83.05)
Ceftaroline fosamil	1	1917	505	16,008,476	16.54 (2.32–117.68)

Trimethoprim	11	1907	9121	15,999,860	10.12 (5.59–18.31)
Sulfadiazine	2	1916	1254	16,007,727	13.32 (3.33–53.38)
Sulfamethoxazole and trimethoprim	33	1885	64,580	15,944,401	4.32 (3.06–6.10)
Erythromycin	17	1901	14,696	15,994,285	9.73 (6.04–15.69)
Clarithromycin	65	1853	26,911	15,982,070	20.83 (16.26–26.69)
Azithromycin	30	1888	38,194	15,970,787	6.64 (4.63–9.53)
Clindamycin	71	1847	27,521	15,981,460	22.32 (17.61–28.30)
Lincomycin	2	1916	235	16,008,746	71.11 (17.67–286.22)
Streptomycin	2	1916	1004	16,007,977	16.64 (4.15–66.69)
Tobramycin	16	1902	19613	15,989,368	6.86 (4.19–11.22)
Gentamicin	22	1896	12,497	15,996,484	14.85 (9.75–22.62)
Amikacin	13	1905	11,707	15,997,274	9.32 (5.40–16.09)
Ofloxacin	7	1911	5281	16,003,700	11.10 (5.28–23.33)
Ciprofloxacin	110	1808	78,326	15,930,655	12.37 (10.21–15.00)
Levofloxacin	71	1847	44,782	15,964,199	13.70 (10.81–17.37)
Moxifloxacin	7	1911	11,955	15,997,026	4.90 (2.33–10.30)
Gatifloxacin	2	1916	1601	16,007,380	10.44 (2.61–41.80)
Vancomycin	164	1754	47,661	15,961,320	31.31 (26.67–36.76)
Polymyxin B	4	1914	985	16,007,996	33.96 (12.71–90.77)
Metronidazole	205	1713	51,725	15,957,256	36.92 (31.93–42.68)
Tinidazole	4	1914	357	16,008,624	93.71 (34.94–251.32)
Nitrofurantoin	1	1917	2650	16,006,331	3.15 (0.44–22.39)
Fosfomycin	2	1916	641	16,008,340	26.07 (6.50–104.54)
Linezolid	25	1893	20,415	15,988,566	10.34 (6.97–15.35)
Daptomycin	6	1912	11,099	15,997,882	4.52 (2.03–10.08)
Tedizolid	1	1917	501	16,008,480	16.67 (2.34–118.63)

Note: Signal detection results for no report of target drug-AE combination (a = 0) is omitted.
Abbreviations: AE, adverse event; CI, confidence interval; ROR, reporting odd ratio.
